# Childhood

**Published:** 1893-01

**Authors:** 


CHILDHOOD: A monthly magazine of all that concerns the welfare of the child. George William Winterburn, M. D., editor; Florence Hull, associate. Published by A. L. Chatterton & Co., New York. One dollar a year, ten cents a number.

With the month of December comes this new magazine for parents, edited by the well-known practitioner and writer, Dr. George W. Winterburn. It is devoted to the physical, intellectual, and ethical interest of the little ones, and as it is needed, will be most welcome. Such a magazine will be appreciated by the intelligent parent whose chief desire is the happiness and welfare of his children. An oft-repeated question is, "What shall we do with and for our children?" and its answer will be found herein.

